# UV radiation and organic matter composition shape bacterial functional diversity in sediments

**DOI:** 10.3389/fmicb.2013.00317

**Published:** 2013-10-28

**Authors:** Ellard R. Hunting, Christopher M. White, Maarten van Gemert, Daan Mes, Eva Stam, Harm G. van, der Geest, Michiel H. S. Kraak, Wim Admiraal

**Affiliations:** ^1^Aquatic Ecology and Ecotoxicology, Institute for Biodiversity and Ecosystem Dynamics, University of AmsterdamAmsterdam, Netherlands; ^2^National Institute for Public Health and the EnvironmentBilthoven, Netherlands

**Keywords:** organic matter quality, UV radiation, benthic bacterial communities, bacterial metabolic diversity

## Abstract

UV radiation and organic matter (OM) composition are known to influence the species composition of bacterioplankton communities. Potential effects of UV radiation on bacterial communities residing in sediments remain completely unexplored to date. However, it has been demonstrated that UV radiation can reach the bottom of shallow waters and wetlands and alter the OM composition of the sediment, suggesting that UV radiation may be more important for sediment bacteria than previously anticipated. It is hypothesized here that exposure of shallow OM-containing sediments to UV radiation induces OM source-dependant shifts in the functional composition of sediment bacterial communities. This study therefore investigated the combined influence of both UV radiation and OM composition on bacterial functional diversity in laboratory sediments. Two different OM sources, labile and recalcitrant OM, were used and metabolic diversity was measured with Biolog GN. Radiation exerted strong negative effects on the metabolic diversity in the treatments containing recalcitrant OM, more than in treatments containing labile OM. The functional composition of the bacterial community also differed significantly between the treatments. Our findings demonstrate that a combined effect of UV radiation and OM composition shapes the functional composition of microbial communities developing in sediments, hinting that UV radiation may act as an important sorting mechanism for bacterial communities and driver for bacterial functioning in shallow waters and wetlands.

## INTRODUCTION

Several studies have demonstrated that UV radiation may affect bacterioplankton communities (e.g., Baldy et al., 2002; Piccini et al., 2009; Zepp et al., 2011), exerting detrimental effects on DNA or extracellular enzymes (e.g., Santos et al., 2012a; for review, see Ruiz-González et al., 2013). In addition, UV radiation may change the chemical composition and palatability of organic matter (OM) by photodegradation (e.g., Engelhaupt et al., 2002; Sulzberger and Durisch-Kaiser, 2009). Such changes in the chemical composition of OM may subsequently cascade toward shifts in bacterial community composition due to the interplay between bacterial resource niches (i.e., the type of substrates that are utilized) and available resources (e.g., Salles et al., 2009). This suggests that the functional composition of bacterial communities may also change when exposed to UV radiation. Several studies indeed demonstrated that photolytic changes of OM can result in altered bacterial production (e.g., Wetzel et al., 1995; Anesio et al., 2000; Tranvik and Bertilsson, 2001) or composition of bacterioplankton communities (Paul et al., 2012), and that UV radiation can induce shifts in the functional composition of bacterioplankton communities (Santos et al., 2012b). 

To date, potential effects of UV radiation on bacterial communities residing in sediments remain completely unexplored. However, UV radiation can penetrate the entire water column and reach the bottom of shallow water bodies and wetlands (e.g., Morris et al., 1995), and alter the OM composition of the sediment (Mayer et al., 2011), suggesting that UV radiation may be more important for sediment bacteria than previously anticipated. It is hypothesized here that exposure of shallow OM-containing sediments to UV radiation induces shifts in the functional composition of sediment bacterial communities. To begin to test this assumption, we compared the effect of UV radiation and dark incubation on bacterial metabolic diversity in sediment microcosms. Since composition of the available OM is one of the main drivers of bacterial community composition (e.g., Hättenschwiler and Vitousek, 2000; Myers et al., 2001; Baldy et al., 2002; Docherty et al., 2006; Kampfraath et al., 2012), and UV radiation differentially affects different OM sources (Benner and Kaiser, 2011), we assessed the effects of UV exposure on bacterial communities on two OM sources that differ in chemical composition.

## MATERIALS AND METHODS

### SEDIMENT MICROCOSMS

Freshly collected stinging nettle, *Urtica dioica*, was used as a labile OM source, and intact peat collected from natural peatlands was used as recalcitrant OM source. UV-absorption spectra of extracts of the used OM sources confirmed that *U. dioica* was a labile OM source (weak absorbance) and peat a recalcitrant source (strong absorbance; data not shown). Both nettle and peat were frozen in liquid nitrogen and thoroughly ground in a pestle and mortar. Quartz sand (0.1–0.5 mm; Dorsilit, Eurogrit, Papendrecht, Netherlands) was mixed with either the labile or recalcitrant OM source (95:5 weight ratio sand:OM source with final dry weight OM concentrations of 0.63 and 0.52% for labile and recalcitrant OM, respectively), and then autoclaved. 5 mL of each prepared sediment was subsequently added to five microcosms, i.e., five replicates per treatment [plastic round vials (Greiner Bio-One, Germany): 27 mm diameter, 5 cm height], resulting in~1 cm sediment layer. Each microcosm received 2 cm of overlying water (Dutch Standard Water, DSW; deionized water with 200 mg/L CaCl_2_.2H_2_O, 180 mg/L MgSO_4_.H_2_O, 100 mg/L NaHCO_3_, and 20 mg/L KHCO_3_; pH = 8.2 ± 0.2). A mixture of sediment pore water and surface water collected from two different natural wetland systems was added as bacterial inoculum.

### EXPERIMENTAL SET UP

Microcosms were incubated at 15°C under a dark:UV regime of 12 h:12 h. We used mercury UV-lamps (Arcadia-D3, Redhill, UK: 160 W; luminous flux 1900 lm) that emits UV radiation of the following intensities: UV-B 1.75 W.m^-^^2^ at 310 nm; and UV-A 10 W.m^-^^2^ at 365 nm. These intensities of UV radiation are commonly registered in temperate areas (Kelly et al., 2003). The duration of the incubation was 5 days. An additional set (*n* = 5) of microcosms of both OM types was incubated in the dark as control. This yielded a total of four treatments, consisting of: (1) labile OM with UV radiation; (2) labile OM in the dark; (3) recalcitrant OM with UV radiation; and (4) recalcitrant OM in the dark. After 5 days, bacterial metabolic diversity was determined as described below.

### COMMUNITY METABOLIC DIVERSITY

Community metabolic diversity (CMD) in the sediment was assessed by community level physiological profiling (CLPP) using Biolog GN microplates containing 95 unique single substrates (Biolog, Inc., Hayward, USA; Garland and Mills, 1991). Biolog GN plates are comprised of simple, common substrates (e.g., sucrose, mallose, and citric acid), and do not include recalcitrant substrates nor specific substrates typical of the OM used in this study. It is therefore impossible to directly relate substrate utilization profiles to the actual functioning of the developed bacterial communities. Nonetheless, the number of substrates used can serve as a proxy of the metabolic diversity of the bacterial community, and differences in utilization profiles indicate that functionally distinct bacterial communities can develop depending on treatment (Garland, 1999; Hunting et al., 2013a). At the end of the experiment (day 5), 1 mL of the sediment top layer with minimal water was sampled with a pipette, diluted 30× with DSW and vortexed. Mineral substrate was allowed to settle and the overlying water containing the porewater bacteria was subsequently distributed over the 96 Biolog GN wells (Hunting et al., 2013b). Plates were incubated for 36 h at 37°C and utilization patterns of 95 different single carbon sources were measured at 490 nm using an automated microplate reader (VERSAmax tunable microplate reader, Molecular Devices, Sunnydale, USA). This data was used to calculate the CMD, i.e., the total number of substrates utilized (Garland, 1997) using a threshold absorbance of 0.15, and analyzed with a two-way ANOVA (analysis of variance) and Tukey’s HSD (honestly significant difference) *post hoc* test. To relate the bacterial functional composition to the four treatments, utilization patterns of the 95 carbon sources were analyzed using a Bray–Curtis-based cluster analysis and a two-way analysis of similarities (ANOSIM; Hammer et al., 2001).

## RESULTS

**FIGURE 1:**
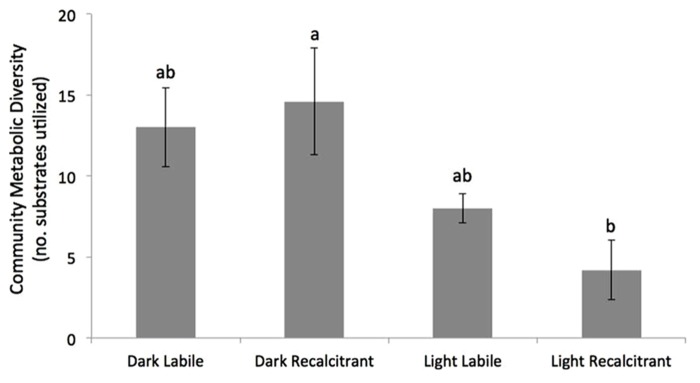
**Mean (±SE) community metabolic diversity (CMD) of the four treatments after 5 days incubation.** Bars with the same letters are not significantly different (two-way ANOVA with Tukey’s HSD *post hoc* test, *n* = 5, *p* < 0.05).

Effects of UV on the metabolic diversity of the different treatments are presented in **Figure 1**. No significant difference was observed between the UV exposure and the control dark incubation in the sediments containing labile OM. In contrast, UV exposure significantly reduced the CMD in the treatments containing recalcitrant OM (two-way ANOVA, Tukey’s HSD, *p* = 0.004; **Figure 1)**.

**FIGURE 2:**
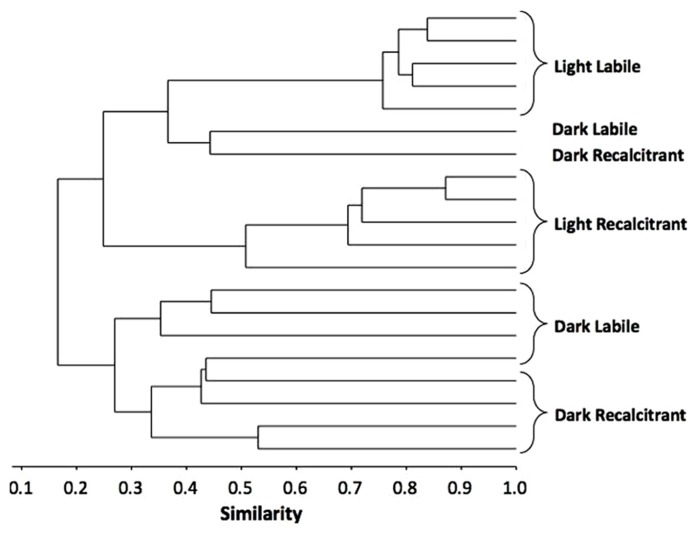
**Bray–Curtis-based dendrogram representing level of similarity between sets of substrates used by the bacterial communities after 5 days incubation at four different conditions**.

In addition to the number of substrates used by the bacterial community, we assessed which set of substrates was used by the bacteria to compare the functional composition of the communities that developed during the incubation. A two-way ANOSIM revealed that the bacterial resource niches differed significantly between treatments depending on both radiation and OM type (two-way ANOSIM: UV radiation *R* = 0.536, *p* = 0.0007; OM *R* = 0.302, *p* = 0.0146, respectively; **Figure 2**), showing that the developed bacterial communities were functionally distinct.

## DISCUSSION

UV radiation diminished the number of organic substrates used by the bacteria and resulted in a dissimilarity of substrate use between bacterial communities. This outcome was most evident when peat was used as substrate. The potential detrimental effects of UV radiation on, e.g., DNA and enzymes (e.g., Santos et al., 2012a; Ruiz-González et al., 2013) are typically held responsible for this negative effect, yet an alternative explanation for our observation may be that compounds liberated during radiation mediated degradation of recalcitrant OM negatively affected some members of the bacterial community. It has been demonstrated that photodegradation of OM creates biologically useful low molecular weight compounds that can promote bacterial production (Biddanda and Cotner, 2003; Obernosterer and Benner, 2004; Anesio et al., 2005), as well as toxic hydrogen peroxide, free radicals and other compounds that inhibit bacterial growth (e.g., Mopper and Zhou, 1990; Scully et al., 1996; Anesio et al., 2000; Tranvik and Bertilsson, 2001). Substituted organic molecules and aromatic products may also form during this process (Mill et al., 1980; Wiegman et al., 2002). Recalcitrant OM, in contrast to labile OM, contains substantial amounts of aromatic compounds (e.g., phenols, lignins, humic acid) that are known to strongly absorb UV-B and to be susceptible to photo degradation (Zepp et al., 1985; Benner and Kaiser, 2011). UV radiation therefore more likely affected the chemical composition of recalcitrant OM than labile OM in this study, explaining why in the present study radiation effects were most prominent on peat. UV radiation thus affects sediment bacteria directly by damaging DNA and extracellular enzymes and indirectly by altering OM composition, an important driver for bacterial functioning (cf. Meyer et al., 1987; Tranvik and Kokalj, 1998; Engelhaupt et al., 2002; Docherty et al., 2006; Köhler et al., 2012).

The results presented here demonstrated that effects of UV radiation on bacterial functional diversity can occur within the top layer of the sediment–water interface, a prominent habitat in mudflats, lakes, streams, and wetlands. UV radiation on sediments has also been shown to degrade particulate OM to dissolved OM (DOM) in coastal sediments, increasing bio-available DOM concentrations and likely fueling heterotrophic bacterial production (Mayer et al., 2011). A large part (more than 50%) of the dead OM-pool becomes trapped in subsurface sediments (Herbst, 1980; Metzler and Smock, 1990). Sediment re-suspension due to wave action (e.g., Sheng and Lick, 1979; Wainright and Hopkinson, 1997) or (in-)faunal locomotion/bioturbation (e.g., Meysman et al., 2006; Hunting et al., 2012) is common in shallow waters and substantially increases the amount of OM exposed to UV. This suggests that UV radiation can be an important sorting mechanism (e.g., Mann and Wetzel, 1995; Santos et al., 2012b) and driver of bacterial functioning in shallow water bodies and wetlands.

## CONCLUSION

This study tested the effect of UV radiation on the functional composition of bacterial communities in shallow aquatic sediments and showed that an interaction between bacterial community metabolism, UV radiation and OM composition occurs at the boundary of sediment and water. Although these results were obtained in simplified systems under laboratory conditions, we conclude that adverse effects of UV radiation on the metabolic diversity are most profound in the presence of recalcitrant OM and that the interaction between UV radiation and OM composition can be an important driver for the functional composition of bacterial communities in shallow benthic environments. This outcome hints that UV radiation is a currently overlooked, but important sorting mechanism and driver of bacterial functioning in shallow waters and wetlands.

## Conflict of Interest Statement

The authors declare that the research was conducted in the absence of any commercial or financial relationships that could be construed as a potential conflict of interest.
